# New Methodology of Designation the Precise Aircraft Position Based on the RTK GPS Solution

**DOI:** 10.3390/s22010021

**Published:** 2021-12-21

**Authors:** Kamil Krasuski, Adam Ciećko, Mieczysław Bakuła, Grzegorz Grunwald, Damian Wierzbicki

**Affiliations:** 1 Institute of Navigation, Polish Air Force University, 08-521 Dęblin, Poland; m.bakula@law.mil.pl; 2 Faculty of Geoengineering, University of Warmia and Mazury in Olsztyn, 10-720 Olsztyn, Poland; a.ciecko@uwm.edu.pl (A.C.); grzegorz.grunwald@uwm.edu.pl (G.G.); 3 Department of Imagery Intelligence, Faculty of Civil Engineering and Geodesy, Military University of Technology, 00-908 Warsaw, Poland; damian.wierzbicki@wat.edu.pl

**Keywords:** RTK-OTF, least square estimation, aircraft position, standard deviation, GPS

## Abstract

The paper presents the results of research on improving the accuracy of aircraft positioning using RTK-OTF (Real Time Kinematic–On The Fly) technique in air navigation. The paper shows a new solution of aircraft positioning for the application of the differential RTK-OTF technique in air navigation. In particular, a new mathematical model is presented which makes it possible to determine the resultant position of an aircraft based on the solution for the method of least squares in a stochastic process. The developed method combines in the process of alignment of GPS (Global Positioning System) observations, three independent solutions of the aircraft position in OTF mode for geocentric coordinates XYZ of the aircraft. Measurement weights as a function of the vector length and the mean vector length error, respectively, were used in the calculations. The applied calculation method makes it possible to determine the resultant position of the aircraft with high accuracy: better than 0.039 m with using the measurement weight as a function of the vector length and better than 0.009 m with the measurement weight as a function of the mean error of the vector length, respectively. In relation to the classical RTK-OTF solution as a model of the arithmetic mean, the proposed method makes it possible to increase the accuracy of determination of the aircraft position by 45–46% using the measurement weight as a function of the vector length, and 86–88% using the measurement weight as a function of the mean error of the vector length, respectively. The obtained test results show that the developed method improves to significantly improve the accuracy of the RTK-OTF solution as a method for determining the reference position in air navigation.

## 1. Introduction

GNSS (Global Navigation Satellite System) satellite technology enables determination of aircraft position using absolute and differential satellite measurements [1]. The use of absolute methods provides aircraft positioning accuracy from the level of ±10 m for SPP (Single Point Positioning) code method [2] to about ±0.1 m for PPP (Precise Point Positioning) method [3,4]. Differential GNSS measurements, on the other hand, achieve an accuracy of ±1 to ±3 m for the DGNSS (Differential GNSS) code differential technique [5] and ±1 cm to ±10 cm for the RTK (Real Time Kinematic) phase differential technique in OTF (On The Fly) mode, respectively [6,7]. It is particularly important to determine the precise reference position of the aircraft during day and night flight operations. The control of position indications determined in relation to the reference position is a key parameter in determining the accuracy of GNSS satellite positioning in aviation. This raises the research problem of how to optimally determine the reference position of the aircraft flight and what geometric configuration of the reference station deployment to use. In previous scientific research conducted around the world, the reference trajectory of an aircraft was determined based on the differential phase RTK-OTF technique [8]. However, in the RTK-OTF solution, the key parameter is the number of reference stations used in the development of GNSS observations and their distribution [9]. Moreover, in RTK measurements, the distance between the reference station and the on-board receiver is affected by the vector measurement error, which varies linearly. The effect of changing this linear trend is especially noticeable for the horizontal coordinates [10]. This error increases as the distance between the reference station and the airborne mobile receiver increases. In contrast, in the RTN (Real Time Network) solution, the final position of the aircraft is determined using corrections from reference stations. In the RTN solution, the basic geometry of the frame of reference stations is a triangle and at least three reference stations [11].

## 2. Related Papers

Research into the application of differential RTK-OTF technology in aviation began as early as the 1990s. There was a need for a GNSS positioning method in air navigation which made it possible to determine the position of an aircraft with particularly high precision. The use of RTK-OTF technology for GPS phase observations ensured the high precision of the determined coordinates of the aircraft. This was important because the aircraft coordinates from the differential RTK-OTF technique were defined as the reference position of a moving object in air navigation. Research on the application of the differential RTK technique was carried out in both real-time and post-processing modes. The paper [12] used the RTK solution in GPS to determine the flight orientation of an aircraft and to determine the YPR (Yaw, Pitch, Roll) angles. In turn, the article [13] shows the operation of Shipboard-Relative GPS (SRGPS) for the procedure of approach and landing in conditions of minimum visibility and using the RTK positioning technique. Whereas work [14] presented the solution of aircraft positioning within the framework of a precision approach and landing category I, taking into account RTK positioning. In work [15], similarly as in publications [13,14], the problem of determining the position of the aircraft with the use of RTK GPS solution in the procedure of precise approach and landing was explored, but this time of category III according to technical standards of ICAO (International Civil Aviation Organization). In addition, work [16] shows the solution of double phase differences (DD-Double Difference) in the RTK method for determining the position of the aircraft. High positioning accuracy, about 0.1 m, on vector lengths up to 200 km was obtained from the study. Interesting research has been carried out in works [17,18], where the solution of the aircraft position with the use of the RTK method was presented, and the problem of determining the phase ambiguity for the LAMBDA (Least-squares AMBiguity Decorrelation Adjustment) method was discussed. Another interesting navigation solution [19], in which the RTK method was used, is the determination of integrity and availability parameters for flight tests. Additionally, paper [19] investigated the effect of phase ambiguity determination on integrity and availability parameters of RTK GPS positioning in aviation. Another publication [20] showed the RTK GPS/GLONASS (Globalnaja Navigatsionnaya Sputnikovaya Sistema) solution for determining aircraft position and basic navigation parameters, such as altitude and separation in airspace. Subsequently, in the publication [21] the RTK GPS method was used to determine approximate values of the centers of projection and external orientation parameters for the purposes of aerial digital aerotriangulation. In paper [22] a scientific study was performed on the effect of RTK-OTF solution on the determination of tropospheric correction during flight test. On the other hand, the paper [23] showed a new criterion for determining phase ambiguity in OTF mode and the impact on precise positioning and orientation of the aircraft for RTK technique. Similar research experiments for determining aircraft orientation and determining YPR angles for RTK GPS solution were presented in work [24]. Within the real-time RTK positioning, an interesting solution was shown in the work [25], where the VRS (Virtual Reference Station) concept was applied to determine the aircraft position for three independent solutions. The positioning accuracy of the aircraft for the VRS concept is higher than 0.1 m. Another article [26] deals with the problem of applying the DD solution for the RTK GPS method for determining the positioning integrity parameters HPL (Horizontal Protection Level) and VPL (Vertical Protection Level) within the GBAS (Ground Based Augmentation System). In turn, in the publication [27], the RTK GPS solution was used as a reference position for determining the accuracy of the PPP (Precise Point Positioning) solution in aviation. In addition, the accuracy of determining the speed and acceleration of the aircraft was also calculated. The DD solution of the RTK GPS method was also used for aircraft flight simulation studies [28]. Based on this, it was determined that the positioning accuracy of the aircraft in the simulation tests is about 0.04 m.

In aviation experiments in Poland, the RTK-OTF differential technique was mainly used to determine the reference position of the flight. Thus, the works [11,29,30,31] showed the results of DGNSS positioning accuracy in relation to the RTK-OTF solution. In turn, works [29,32,33,34,35] presented position error results for the SPP (Single Point Positioning) positioning method in air navigation. Subsequently, papers [32,34,36,37] present results of EGNOS (European Geostationary Navigation Overlay Service) solution in relation to the reference position of the flight determined from the differential RTK-OTF technique. Furthermore, in papers [38,39] the results of PPP positioning were compared with the RTK-OTF solution. Then, in the work [40], the model of the resultant flight speed of the aircraft was calculated and the results were compared with the RTK-OTF precision solution. In publication [41], the results of determining the XYZ geocentric coordinates of the aircraft from three independent RTK solutions in OTF mode were presented. The coordinate difference between the different RTK-OTF solutions is less than 0.19 m. Another work [42] shows the results of determining the ellipsoid of the point position error for BLh geodetic coordinates determined from the differential RTK-OTF technique. The results of the point position error ellipsoid parameter of 0.03 m were obtained in the calculations.

On the basis of the collected publications [11,12,13,14,15,16,17,18,19,20,21,22,23,24,25,26,27,28,29,30,31,32,33,34,35,36,37,38,39,40,41,42], it can be seen that a lot of research work concerned the determination of positioning accuracy using the RTK-OTF technique. In order to better describe the research carried out in the works [11,12,13,14,15,16,17,18,19,20,21,22,23,24,25,26,27,28,29,30,31,32,33,34,35,36,37,38,39,40,41,42], Table 1 presents a short summary of the obtained results of the accuracy parameter. The results in Table 1 show that in many research papers [11,20,22,23,25,29,33,34,36,41,42], the positioning accuracy for the vertical component was lower than 0.1 m. In [14,15,16,20,22,23,25,28,29,33,34,36,41,42] it can be seen that the positioning accuracy was higher than 0.1 m and mainly related to horizontal components of the aircraft position or all three component items.

On the basis of the presented state analysis based on the works [11,12,13,14,15,16,17,18,19,20,21,22,23,24,25,26,27,28,29,30,31,32,33,34,35,36,37,38,39,40,41,42], it can be said that the application of the differential RTK-OTF technique is most reasonable and necessary. More so, as shown in works [11,12,13,14,15,16,17,18,19,20,21,22,23,24,25,26,27,28,29,30,31,32,33,34,35,36,37,38,39,40,41,42], the scope of research on the use of differential RTK-OTF technique in air navigation is wide and quite extensive. Moreover, it can be stated that this positioning method is in the circle of interest of many research centers in the world. The analysis of the state of the art [11,12,13,14,15,16,17,18,19,20,21,22,23,24,25,26,27,28,29,30,31,32,33,34,35,36,37,38,39,40,41,42] shows that the RTK-OTF method has the most applications in the area of aircraft positioning in air navigation. Therefore, the present work will also concern the area of RTK GPS positioning and, in particular, propose a new computational strategy to determine the resultant position of an aircraft in a stochastic process within the RTK-OTF differential technique. In this paper, a mathematical algorithm is applied to determine the resultant aircraft position based on the alignment of aircraft position coordinates using the least squares method and taking into account measurement weights. The input coordinates used in the calculations were determined independently for a single baseline within the RTK technique. In the calculations, weighting was applied as a function of the inverse of the distance of the reference station-GNSS receiver vector and as a function of the inverse of the square of the mean error of the baseline distance measurement. Finally, the coordinates of the aircraft are determined as the resultant position of the aircraft determined in a stochastic process on the basis of three independent RTK determinations in OTF mode. The presented new computational strategy enables the effective improvement of aircraft positioning accuracy using RTK-OTF technique in air navigation.

Our main contribution to research on the application of RTK-OTF technology in air navigation concerns:

- development of a mathematical algorithm to align aircraft coordinates from three independent RTK position determinations in OTF mode,
- the use of the least squares method for the calculation of the proposed calculation algorithm,
- the use of measurement weights in a stochastic process to develop aircraft coordinate results,
- carry out an accuracy analysis for the proposed calculation strategy,
- demonstrate that the computational algorithm used is superior to an arithmetic mean model and a mathematical model based on two independent RTK solutions in OTF mode.

## 3. Research Method

### 3.1. RTK-OTF Positioning Model for Single Baseline

The basic observation equation for the differential RTK-OTF technique in GPS can be written as follows [43,44,45]:

(1) $$\{\begin{array}{c} \nabla \Delta \lambda_{1}\cdot \phi_{AB,1}^{ij}=\rho_{AB}^{ij}-\nabla \Delta I_{AB,1}^{ij}+\nabla \Delta T_{AB}^{ij}+\nabla \Delta \lambda_{1}\cdot N_{AB,1}^{ij}+\varpi \\ \nabla \Delta \lambda_{2}\cdot \phi_{AB,2}^{ij}=\rho_{AB}^{ij}-\nabla \Delta I_{AB,2}^{ij}+\nabla \Delta T_{AB}^{ij}+\nabla \Delta \lambda_{2}\cdot N_{AB,2}^{ij}+\varepsilon \end{array}$$

where:

∇—symbol of double difference for the phase measurements, allows the comparison of phase measurements from two tracking satellites by two receivers,

Δ—symbol of single difference for the phase measurements, allows setting the difference of the phase measurements from two tracking satellites by one receiver,

$\lambda_{1}$—wavelength on L1 frequency in GPS system,

$\lambda_{2}$—wavelength on L2 frequency in GPS system,

$\phi_{AB,1}^{ij}$—double difference for phase observations (given in cycles) on $A\vec{B}$ vector between the satellites i and j on L1 frequency in GPS system,

$\phi_{AB,2}^{ij}$—double difference for phase observations (given in cycles) on $A\vec{B}$ vector between the satellites i and j on L2 frequency in GPS system,

$\rho_{AB}^{ij}$—geometric distance of $A\vec{B}$ vector (given in geocentric *XYZ* coordinates),

$I_{AB,1}^{ij}$—value of ionosphere delay on L1 frequency for double difference for phase observations,

$I_{AB,2}^{ij}$—value of ionosphere delay on L2 frequency for double difference for phase observations,

$I_{AB,2}^{ij}=\gamma \cdot I_{AB,1}^{ij}$, relation of the ionosphere delay on L1 and L2 frequency,

$\gamma ={\left(\frac{f_{1}}{f_{2}}\right)}^{2}$,

$f_{1}$—L1 frequency in GPS system,

$f_{2}$—L2 frequency in GPS system,

$T_{AB}^{ij}$—troposphere delay value for double difference for phase observations,

$N_{AB,1}^{ij}$—phase ambiguity value on L1 frequency for double difference for phase observations,

$N_{AB,2}^{ij}$—phase ambiguity value on L2 frequency for double difference for phase observations,

ϖ—measurement noise (multipath and receiver noise) for code observations,

ε—measurement noise (multipath and receiver noise) for phase observations.

The parameter $\rho_{AB}^{ij}$ is in turn expressed using a mathematical formula:

(2) $$\rho_{AB}^{ij}=\sqrt{{\left(X_{A}-X_{sat}^{i}\right)}^{2}+{\left(Y_{A}-Y_{sat}^{i}\right)}^{2}+{\left(Z_{A}-Z_{sat}^{i}\right)}^{2}}-\sqrt{{\left(X_{B}-X_{sat}^{j}\right)}^{2}+{\left(Y_{B}-Y_{sat}^{j}\right)}^{2}+{\left(Z_{B}-Z_{sat}^{j}\right)}^{2}}$$

where:

$\left(X_{A},Y_{A},Z_{A}\right)$—coordinates of the reference station,

$\left(X_{B},Y_{B},Z_{B}\right)$—coordinates of the aircraft to be determined,

$\left(X_{sat}^{i},Y_{sat}^{i},Z_{sat}^{i}\right)$—coordinates of the GPS *i*-th satellite,

$\left(X_{sat}^{j},Y_{sat}^{j},Z_{sat}^{j}\right)$—coordinates of the GPS *j*-th satellite.

Equation (1) is used to determine the position of an aircraft for a single baseline (vector) geometry in a GPS satellite system.

### 3.2. RTK-OTF Positioning Model for Multiple Baselines

The positioning model for the geometry of a system consisting of several vectors can be written in turn as follows:

(3) $$\{\begin{array}{c} X_{final}=\frac{\sum X_{B}^{s}}{\sum s}\\ Y_{final}=\frac{\sum Y_{B}^{s}}{\sum s}\\ Z_{final}=\frac{\sum Z_{B}^{s}}{\sum s} \end{array}$$

where:

s—a single baseline number, s∈(1,2,…,S),

∑s—sum of all baselines,

S—the number of the last baseline in the vector geometry,

$\left(X_{final},Y_{final},Z_{final}\right)$—the resultant position of the aircraft,

$\sum X_{B}^{s}$—sum of coordinates along the *X* axis based on the geometry of the vectors,

$\sum Y_{B}^{s}$—sum of coordinates along the *Y* axis based on the geometry of the vectors,

$\sum Z_{B}^{s}$—sum of coordinates along the *Z* axis based on the geometry of the vectors.

In a multi-vector RTK-OTF positioning model, the aircraft coordinates are determined for a single baseline according to Equation (1), and then the resultant value of the aircraft position is calculated separately for each *XYZ* component. The mathematical model is based on the arithmetic mean method according to Equation (3). The aircraft coordinate values along the *XYZ* axis are summed and then the resultant value is calculated. The mathematical model in Equation (3) is devoid of measurement weights, so the precision of aircraft coordinates determined from a single baseline is assumed to be equal.

### 3.3. New Approach for RTK-OTF Technique Based on Multiple Baseline

The paper proposes a new strategy for determining the resultant position of an aircraft for the differential RTK-OTF technique based on a system of vector geometries. In the analyzed case the resultant position of the plane is determined by the condition of minimum geometry of vectors:

(4) $$k\leq 3\cdot s_{\min}$$

where:

k—number of parameters to be determined, in this case the *XYZ* coordinates of the aircraft, k=3,

$s_{\min}=1$, single baseline,

$3\cdot s_{\min}=3$.

On the basis of Formula (4) it can be concluded that at least 3 vectors in space between the reference stations and the on-board GPS receiver are needed to determine the resultant *XYZ* position of the aircraft from the differential RTK-OTF technique. For such a geometrical construction, the observation model for the determination of the resultant aircraft position can be written in the general case as below:

(5) $$\{\begin{array}{c} X_{B,s}=X_{A,s}+dX_{A,s-B,s}\\ Y_{B,s}=Y_{A,s}+dY_{A,s-B,s}\\ Z_{B,s}=Z_{A,s}+dZ_{A,s-B,s} \end{array}$$

where:

$\left(X_{A,s},Y_{A,s},Z_{A,s}\right)$—coordinates of the reference station for a given baseline,

$\left(X_{B,s},Y_{B,s},Z_{B,s}\right)$—coordinates of the aircraft determined for a given base line,

$\left(dX_{A,s-B,s},dY_{A,s-B,s},dZ_{A,s-B,s}\right)$—coordinate difference.

The detailed notation of Equation (5) for 3 baselines will be as follows:

(6) $$\begin{array}{l} \{\begin{array}{c} X_{B,1}=X_{A,1}+dX_{A,1-B,1}\\ Y_{B,1}=Y_{A,1}+dY_{A,1-B,1}\\ Z_{B,1}=Z_{A,1}+dZ_{A,1-B,1} \end{array}\\ \{\begin{array}{c} X_{B,2}=X_{A,2}+dX_{A,2-B,2}\\ Y_{B,2}=Y_{A,2}+dY_{A,2-B,2}\\ Z_{B,2}=Z_{A,2}+dZ_{A,2-B,2} \end{array}\\ \{\begin{array}{c} X_{B,3}=X_{A,3}+dX_{A,3-B,3}\\ Y_{B,3}=Y_{A,3}+dY_{A,3-B,3}\\ Z_{B,3}=Z_{A,3}+dZ_{A,3-B,3} \end{array} \end{array}$$

where:

$\left(X_{A,1},Y_{A,1},Z_{A,1}\right)$—coordinates of the reference station for given baseline 1,

$\left(X_{B,1},Y_{B,1},Z_{B,1}\right)$—coordinates of the aircraft determined for a given baseline 1,

$\left(dX_{A,1-B,1},dY_{A,1-B,1},dZ_{A,1-B,1}\right)$—the coordinate difference between the reference station position and the aircraft position for baseline 1,

$\left(X_{A,2},Y_{A,2},Z_{A,2}\right)$—coordinates of the reference station for a given baseline 2,

$\left(X_{B,2},Y_{B,2},Z_{B,2}\right)$—coordinates of the aircraft determined for a given baseline 2,

$\left(dX_{A,2-B,2},dY_{A,2-B,2},dZ_{A,2-B,2}\right)$—the coordinate difference between the reference station position and the aircraft position for baseline 2,

$\left(X_{A,3},Y_{A,3},Z_{A,3}\right)$—coordinates of the reference station for a given baseline 3,

$\left(X_{B,3},Y_{B,3},Z_{B,3}\right)$—coordinates of the aircraft determined for a given baseline 3,

$\left(dX_{A,3-B,3},dY_{A,3-B,3},dZ_{A,3-B,3}\right)$—the coordinate difference between the reference station position and the aircraft position for baseline 3.

In summary, the coordinates $\left(X_{B,1},Y_{B,1},Z_{B,1}\right)$, $\left(X_{B,2},Y_{B,2},Z_{B,2}\right)$ and $\left(X_{B,3},Y_{B,3},Z_{B,3}\right)$ should be equal to each other:

(7) $$\{\begin{array}{c} X_{B,1}=X_{B,2}=X_{B,3}=X_{B}\\ Y_{B,1}=Y_{B,2}=Y_{B,3}=Y_{B}\\ Z_{B,1}=Z_{B,2}=Z_{B,3}=Z_{B} \end{array}$$

where:

$\left(X_{B},Y_{B},Z_{B}\right)$—final resultant coordinates of the aircraft.

However, due to the distribution of reference stations, vector measurement errors and the dynamics of the aircraft flight, the condition in Equation (7) is not fulfilled. Therefore, the aircraft coordinates from the observation Equation (6) can be aligned by a stochastic process using the least squares method [46]:

(8) A⋅Q−l=v

where:

A—plan matrix,

Q—determined increments to the aircraft coordinates,

l—vector with difference between measurements and modeled parameters,

v—correction vector.

Equation (6) can now be represented by Expression (8):

(9) $$\begin{array}{l} \{\begin{array}{c} (X_{B,0}+\delta X_{B,0})-(X_{A,1}+dX_{A,1-B,1})=v_{X_{B,1}}\\ (Y_{B,0}+\delta Y_{B,0})-(Y_{A,1}+dY_{A,1-B,1})=v_{Y_{B,1}}\\ (Z_{B,0}+\delta Z_{B,0})-(Z_{A,1}+dZ_{A,1-B,1})=v_{Z_{B,1}} \end{array}\\ \{\begin{array}{c} (X_{B,0}+\delta X_{B,0})-(X_{A,2}+dX_{A,2-B,2})=v_{X_{B,2}}\\ (Y_{B,0}+\delta Y_{B,0})-(Y_{A,2}+dY_{A,2-B,2})=v_{Y_{B,2}}\\ (Z_{B,0}+\delta Z_{B,0})-(Z_{A,2}+dZ_{A,2-B,2})=v_{Z_{B,2}} \end{array}\\ \{\begin{array}{c} (X_{B,0}+\delta X_{B,0})-(X_{A,3}+dX_{A,3-B,3})=v_{X_{B,3}}\\ (Y_{B,0}+\delta Y_{B,0})-(Y_{A,3}+dY_{A,3-B,3})=v_{Y_{B,3}}\\ (Z_{B,0}+\delta Z_{B,0})-(Z_{A,3}+dZ_{A,3-B,3})=v_{Z_{B,3}} \end{array} \end{array}$$

$\left(X_{B,0},Y_{B,0},Z_{B,0}\right)$—approximate coordinates of the aircraft determined as the arithmetic mean of 3 baselines,

$X_{B,0}=\frac{X_{B,1}+X_{B,2}+X_{B,3}}{3}$,

$Y_{B,0}=\frac{Y_{B,1}+Y_{B,2}+Y_{B,3}}{3}$,

$Z_{B,0}=\frac{Z_{B,1}+Z_{B,2}+Z_{B,3}}{3}$,

$\left(\delta X_{B,0},\delta Y_{B,0},\delta Z_{B,0}\right)$—determined increments to the approximate position of the aircraft,

$\left(v_{X_{B,1}},v_{X_{B,2}},v_{X_{B,3}}\right)$—corrections along the *X* axis,

$\left(v_{Y_{B,1}},v_{Y_{B,2}},v_{Y_{B,3}}\right)$—corrections along the *Y* axis,

$\left(v_{Z_{B,1}},v_{Z_{B,2}},v_{Z_{B,3}}\right)$—corrections along the *Z* axis.

Successively in Equation (9), the unknown parameters will be separated from the model according to Equation (8):

(10) $$\begin{array}{l} \{\begin{array}{c} \delta X_{B,0}-(X_{A,1}+dX_{A,1-B,1}-X_{B,0})=v_{X_{B,1}}\\ \delta Y_{B,0}-(Y_{A,1}+dY_{A,1-B,1}-Y_{B,0})=v_{Y_{B,1}}\\ \delta Z_{B,0}-(Z_{A,1}+dZ_{A,1-B,1}-Z_{B,0})=v_{Z_{B,1}} \end{array}\\ \{\begin{array}{c} \delta X_{B,0}-(X_{A,2}+dX_{A,2-B,2}-X_{B,0})=v_{X_{B,2}}\\ \delta Y_{B,0}-(Y_{A,2}+dY_{A,2-B,2}-Y_{B,0})=v_{Y_{B,2}}\\ \delta Z_{B,0}-(Z_{A,2}+dZ_{A,2-B,2}-Z_{B,0})=v_{Z_{B,2}} \end{array}\\ \{\begin{array}{c} \delta X_{B,0}-(X_{A,3}+dX_{A,3-B,3}-X_{B,0})=v_{X_{B,3}}\\ \delta Y_{B,0}-(Y_{A,3}+dY_{A,3-B,3}-Y_{B,0})=v_{Y_{B,3}}\\ \delta Z_{B,0}-(Z_{A,3}+dZ_{A,3-B,3}-Z_{B,0})=v_{Z_{B,3}} \end{array} \end{array}$$

In matrix form, Equation (10) can be written as follows:

(11) $$\underset{A}{\underset{︸}{\left[\begin{array}{ccc} 1 & 0 & 0\\ 0 & 1 & 0\\ 0 & 0 & 1\\ 1 & 0 & 0\\ 0 & 1 & 0\\ 0 & 0 & 1\\ 1 & 0 & 0\\ 0 & 1 & 0\\ 0 & 0 & 1 \end{array}\right]}}\cdot \underset{Q}{\underset{︸}{\left[\begin{array}{c} \delta X_{B,0}\\ \delta Y_{B,0}\\ \delta Z_{B,0} \end{array}\right]}}-\underset{l}{\underset{︸}{\left[\begin{array}{c} X_{A,1}+dX_{A,1-B,1}-X_{B,0}\\ Y_{A,1}+dY_{A,1-B,1}-Y_{B,0}\\ Z_{A,1}+dZ_{A,1-B,1}-Z_{B,0}\\ X_{A,2}+dX_{A,2-B,1}-X_{B,0}\\ Y_{A,2}+dY_{A,2-B,1}-Y_{B,0}\\ Z_{A,2}+dZ_{A,2-B,1}-Z_{B,0}\\ X_{A,3}+dX_{A,3-B,3}-X_{B,0}\\ Y_{A,3}+dY_{A,3-B,3}-Y_{B,0}\\ Z_{A,3}+dZ_{A,3-B,3}-Z_{B,0} \end{array}\right]}}=\underset{v}{\underset{︸}{\left[\begin{array}{c} v_{X_{B,1}}\\ v_{Y_{B,1}}\\ v_{Z_{B,1}}\\ v_{X_{B,2}}\\ v_{Y_{B,2}}\\ v_{Z_{B,2}}\\ v_{X_{B,3}}\\ v_{Y_{B,3}}\\ v_{Z_{B,3}} \end{array}\right]}}$$

Equation (11) is solved using a normal equations solution as follows [47]:

(12) $$\begin{array}{l} N=A^{T}\cdot P\cdot A\\ L=A^{T}\cdot P\cdot l\\ Q=N^{-1}\cdot L \end{array}$$

where:

N—the matrix of a normal equation’s solution,

L—misclosure vector,

P—matrix of weights.

The measurement weight in Equation (13) takes the form:

(13) $$P=\frac{1}{d}\vee P=\frac{1}{md^{2}}$$

where:

d—baseline length (vector),

md—mean error of measurement of the vector.

The measurement weights in Equation (13) take the form respectively:

- case I:

(14) $$P=\frac{1}{d}=\left[\begin{array}{ccccccccc} \frac{1}{d_{A,1-B,1}} & 0 & 0 & 0 & 0 & 0 & 0 & 0 & 0\\ 0 & \frac{1}{d_{A,1-B,1}} & 0 & 0 & 0 & 0 & 0 & 0 & 0\\ 0 & 0 & \frac{1}{d_{A,1-B,1}} & 0 & 0 & 0 & 0 & 0 & 0\\ 0 & 0 & 0 & \frac{1}{d_{A,2-B,2}} & 0 & 0 & 0 & 0 & 0\\ 0 & 0 & 0 & 0 & \frac{1}{d_{A,2-B,2}} & 0 & 0 & 0 & 0\\ 0 & 0 & 0 & 0 & 0 & \frac{1}{d_{A,2-B,2}} & 0 & 0 & 0\\ 0 & 0 & 0 & 0 & 0 & 0 & \frac{1}{d_{A,3-B,3}} & 0 & 0\\ 0 & 0 & 0 & 0 & 0 & 0 & 0 & \frac{1}{d_{A,3-B,3}} & 0\\ 0 & 0 & 0 & 0 & 0 & 0 & 0 & 0 & \frac{1}{d_{A,3-B,3}} \end{array}\right]$$

where:

$\frac{1}{d_{A,1-B,1}}$—measurement weight for the baseline A,1−B,1 (reference station No. 1—aircraft),

$\frac{1}{d_{A,2-B,2}}$—measurement weight for the baseline A,2−B,2 (reference station No. 2—aircraft),

$\frac{1}{d_{A,3-B,3}}$—measurement weight for the baseline A,3−B,3 (reference station No. 3—aircraft),

- case II:

(15) $$P=\frac{1}{md^{2}}=\left[\begin{array}{ccccccccc} \frac{1}{md_{A,1-B,1}^{2}} & 0 & 0 & 0 & 0 & 0 & 0 & 0 & 0\\ 0 & \frac{1}{md_{A,1-B,1}^{2}} & 0 & 0 & 0 & 0 & 0 & 0 & 0\\ 0 & 0 & \frac{1}{md_{A,1-B,1}^{2}} & 0 & 0 & 0 & 0 & 0 & 0\\ 0 & 0 & 0 & \frac{1}{md_{A,2-B,2}^{2}} & 0 & 0 & 0 & 0 & 0\\ 0 & 0 & 0 & 0 & \frac{1}{md_{A,2-B,2}^{2}} & 0 & 0 & 0 & 0\\ 0 & 0 & 0 & 0 & 0 & \frac{1}{md_{A,2-B,2}^{2}} & 0 & 0 & 0\\ 0 & 0 & 0 & 0 & 0 & 0 & \frac{1}{md_{A,3-B,3}^{2}} & 0 & 0\\ 0 & 0 & 0 & 0 & 0 & 0 & 0 & \frac{1}{md_{A,3-B,3}^{2}} & 0\\ 0 & 0 & 0 & 0 & 0 & 0 & 0 & 0 & \frac{1}{md_{A,3-B,3}^{2}} \end{array}\right]$$

where:

$\frac{1}{md_{A,1-B,1}^{2}}$—measurement weight for the baseline A,1−B,1 (reference station No. 1—aircraft),

$\frac{1}{md_{A,2-B,2}^{2}}$—measurement weight for the baseline A,2−B,2 (reference station No. 2—aircraft),

$\frac{1}{md_{A,3-B,3}^{2}}$—measurement weight for the baseline A,3−B,3 (reference station No. 3—aircraft).

The corrections, the mean error of the correction, the mean errors of the estimated coordinates, the standard deviation of the corrections are determined successively and a global Chi-square statistical test is performed as follows [48,49]:

(16) $$\begin{array}{l} v=A\cdot Q-l\\ m0=\sqrt{\frac{\left[P\cdot v\cdot v\right]}{n-k}}\\ CQ=m0^{2}\cdot N^{-1}\\ mQ=diag(\sqrt{CQ})={\left[mX\;mY\;mZ\right]}^{T}\\ StdX=\sqrt{\frac{\left[P\cdot v_{X_{B,1}}\cdot v_{X_{B,1}}+P\cdot v_{X_{B,2}}\cdot v_{X_{B,2}}+P\cdot v_{X_{B,3}}\cdot v_{X_{B,3}}\right]}{n-1}}\\ StdY=\sqrt{\frac{\left[P\cdot v_{Y_{B,1}}\cdot v_{Y_{B,1}}+P\cdot v_{Y_{B,2}}\cdot v_{Y_{B,2}}+P\cdot v_{Y_{B,3}}\cdot v_{Y_{B,3}}\right]}{n-1}}\\ StdZ=\sqrt{\frac{\left[P\cdot v_{Z_{B,1}}\cdot v_{Z_{B,1}}+P\cdot v_{Z_{B,2}}\cdot v_{Z_{B,2}}+P\cdot v_{Z_{B,3}}\cdot v_{Z_{B,3}}\right]}{n-1}}\\ \sum Pvv\leq \chi_{f,1-\alpha }^{2} \end{array}$$

where:

m0—mean error,

*n*—number of observations, n=9 for each measurement epoch,

CQ—variance-covariance matrix,

mQ—vector of mean errors of the determined coordinates,

(mX,mY,mZ)—mean errors of the determined *XYZ* coordinates,

(StdX,StdY,StdZ)—standard deviation of the determined *XYZ* coordinates,

$\chi_{f,1-\alpha }^{2}$—the table value of the Chi-square test,

f=n−k=6—number of degrees of freedom,

1−α=0.95—confidence level.

The computational process for the new RTK-OTF positioning strategy for baselines ends with Equation (16). The final resultant aircraft coordinates for the proposed computational strategy can be determined by the formula [50]:

(17) $$\{\begin{array}{c} X_{B}=X_{B,0}+\delta X_{B,0}\\ Y_{B}=Y_{B,0}+\delta Y_{B,0}\\ Z_{B}=Z_{B,0}+\delta Z_{B,0} \end{array}$$

## 4. Research Test

The research methodology was tested during the realization of a flight experiment with a Cessna 172 aircraft around the EPDE (Europe Poland Deblin) military airport in Deblin. The research test lasted from 14:32:09 to 15:00:04. The flight experiment was performed using a Cessna 172 aircraft, belonging to the air fleet of the Military University of Aviation in Dęblin. A Topcon HiperPro dual-frequency receiver was installed in the cockpit of the Cessna 172 aircraft, which recorded code-phase GPS observations with an interval of 1 s. In addition, GPS reference stations were used to realize the geometry of the base vector system. Namely, at the EPDE airport in Dęblin, a REF1 physical station is installed for permanent monitoring of GPS observations. Moreover, thanks to POZGEO service of ASG-EUPOS (Active Geodetic Network EUPOS) [51], two virtual reference stations VirA and VirB were generated. The geometry of the 3 GPS base stations allowed for the construction of vectors between the individual stations and the aircraft. A total of 3 baselines were created:

- reference station REF1–Cessna 172 aircraft (reference A,1–B,1),
- virtual station VirA–Cessna 172 aircraft (identification A,2–B,2),
- virtual station VirB–Cessna 172 aircraft (identification A,3–B,3).

**Figure 1:**
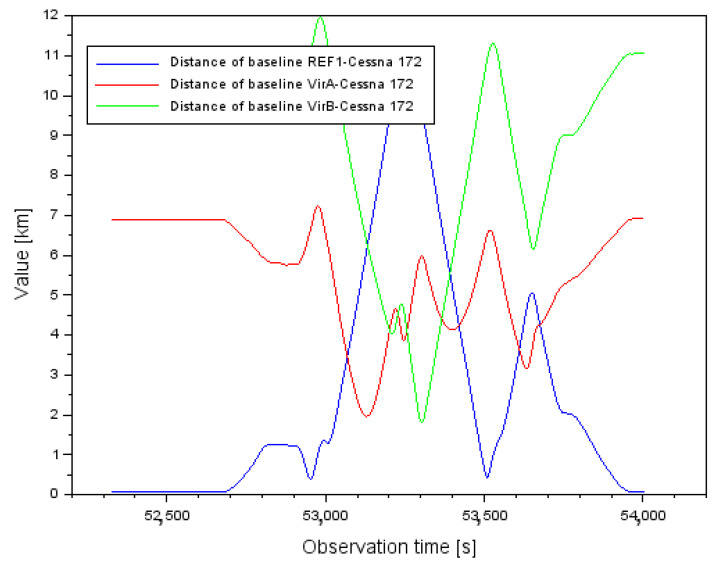
Distance of baselines between each GPS reference station and Cessna 172 aircraft.

Figure 1 shows the results of the vector lengths d between the different GPS reference stations and the position of the Cessna 172 aircraft. In the case of the REF1-Cessna 172 vector, the baseline length varied from 0.058 km to 10.305 km. Conversely, for the VirA-Cessna 172 vector, the baseline length varied from 1.960 km to 7.230 km. In contrast, for the VirB-Cessna 172 vector, the baseline length varied from 1.811 km to 11.969 km.

**Figure 2:**
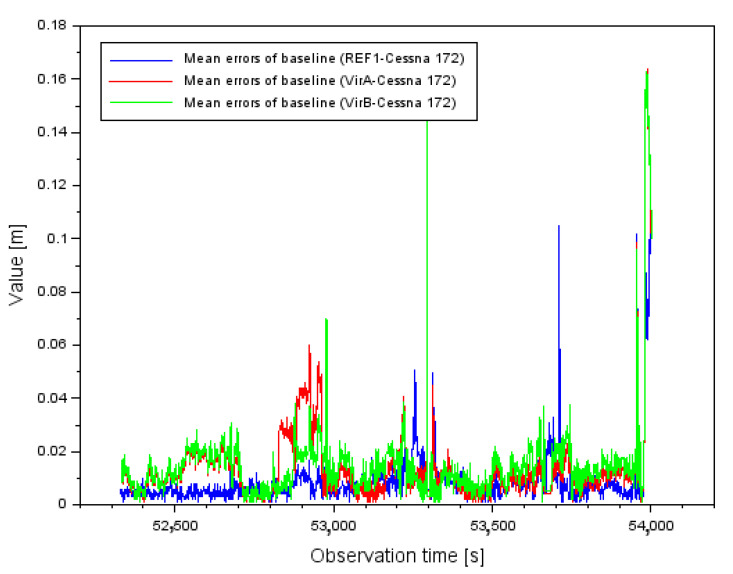
Mean 3D errors for each baseline.

**Figure 3:**
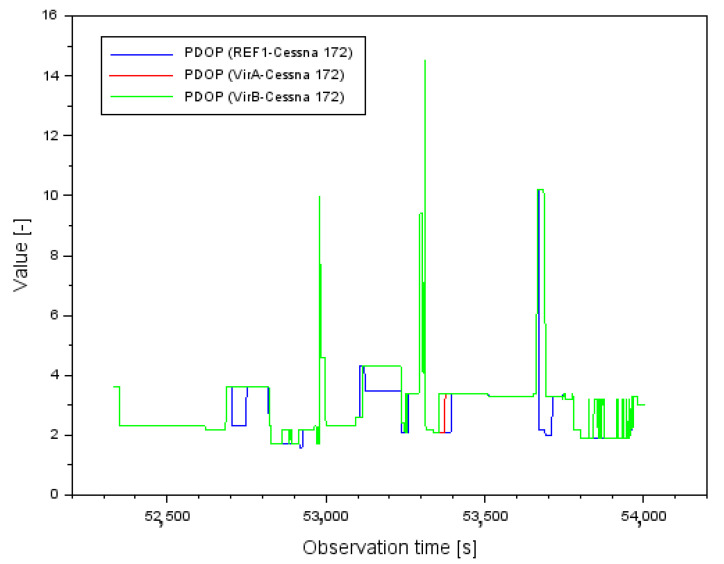
The values of PDOP for each baseline.

**Figure 4:**
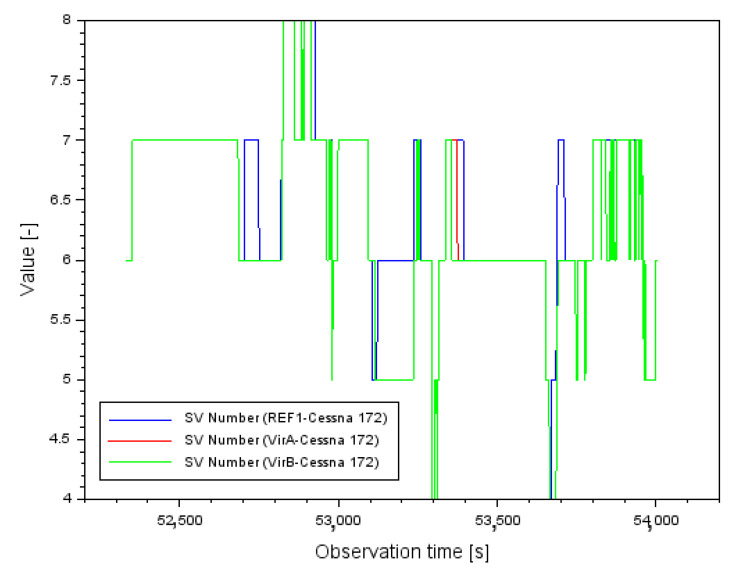
The SV (Satellite Vehicle) number for each baseline.

Figure 2 shows the results of the mean errors of the vector length measurements d between individual GPS reference stations and the position of the Cessna 172 aircraft. For the REF1-Cessna 172 vector, the mean error of the baseline length varied from 0.001 m to 0.142 m. In contrast, for the VirA-Cessna 172 vector, the mean baseline length error varied from 0.001 m to 0.164 m. In contrast, for the VirB-Cessna 172 vector, the mean baseline length error varied from 0.001 m to 0.163 m. When analyzing the results in Figure 2, it should be noted that for single measurement epochs the values of the mean errors of the vector length determination are quite large. In order to better understand this problem, it is worth analyzing the results of the PDOP (Position DOP) coefficients [52], shown in Figure 3. Analyzing the results in Figure 3, it can be said that for most of the flight duration, the PDOP values were less than 4, which corresponds to good observation conditions. However, for a few measurement epochs, outlier results of the PDOP geometric coefficients can be observed. These PDOP outlier results occur in the same measurement epochs as the large jumps in the mean error values in Figure 2. For the PDOP coefficients, outlier results of 10 and 14.5 occur, which is noticeable quite well in Figure 3 for the middle phase of the flight. The jumps in the mean distance errors in the final phase of flight still need to be explained. For this purpose, it is useful to look at the results shown in Figure 4. Figure 4 shows the change in number of tracked GPS satellites for each vector between the reference station and the on-board GPS receiver. Regarding the final stage of the flight, the change in the number of tracked GPS satellites is very dynamic. It is possible to observe 7 tracked GPS satellites, then 6, then 5 for a longer part of the flight, and then there is a jump to 6 again. Such a dynamic variation in time of tracking GPS satellites for the geometry of the 3 vectors obviously causes deterioration of the results of the average distance measurement errors, so that the values above 0.160 m in Figure 2 are noticeable. Looking objectively at the results shown in Figure 2, Figure 3 and Figure 4, it can be seen that the change in the number of tracked GPS satellites and the change in PDOP coefficients affect the geometry of the 3 vector system by changing the values of the average baseline measurement errors. For navigation calculations, especially for the RTK or DGNSS method where baseline vectors are considered, numerical analyses for the parameters presented in Figure 2, Figure 3 and Figure 4 are therefore necessary.

The *XYZ* reference coordinates of the GPS base stations are respectively:

- REF1: *X* = 3,687,932.2628 m, *Y* = 1,480,229.9043 m, *Z* = 4,972,325.4585 m;
- VirA: *X* = 3,684,534.5896 m, *Y* = 1,486,155.7033 m, *Z* = 4,973,133.0806 m;
- VirB: *X* = 3,689,054.1177 m, *Y* = 1,490,474.6121 m, *Z* = 4,968,518.9789 m.

**Figure 5:**
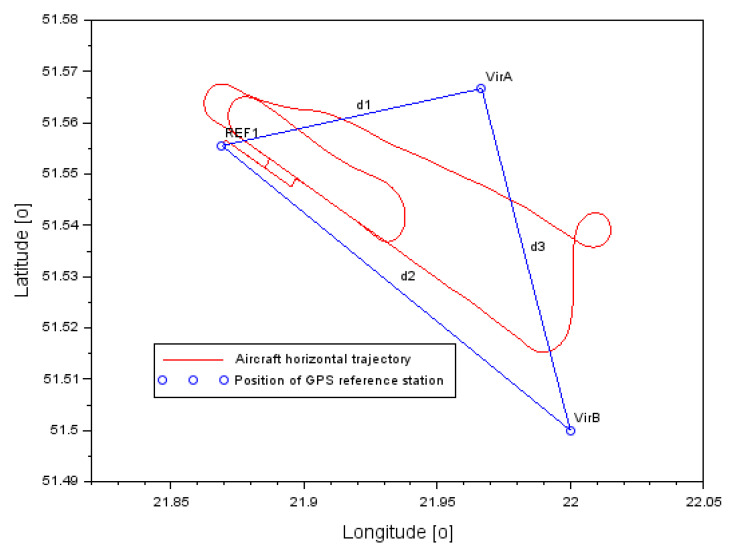
Horizontal trajectory of Cessna 172 aircraft.

Figure 5 shows the flight trajectory of the Cessna 172 aircraft along with the location of the GPS base stations. The position of the aircraft and the GPS base stations is expressed in BL ellipsoidal coordinates (B-Latitude, L-Longitude). In Figure 5, the distances between the GPS base stations are marked as:

- d1: distance between stations REF1 and VirA,
- d2: distance between stations REF1 and VirB,
- d3: distance between VirA and VirB stations.

**Figure 6:**
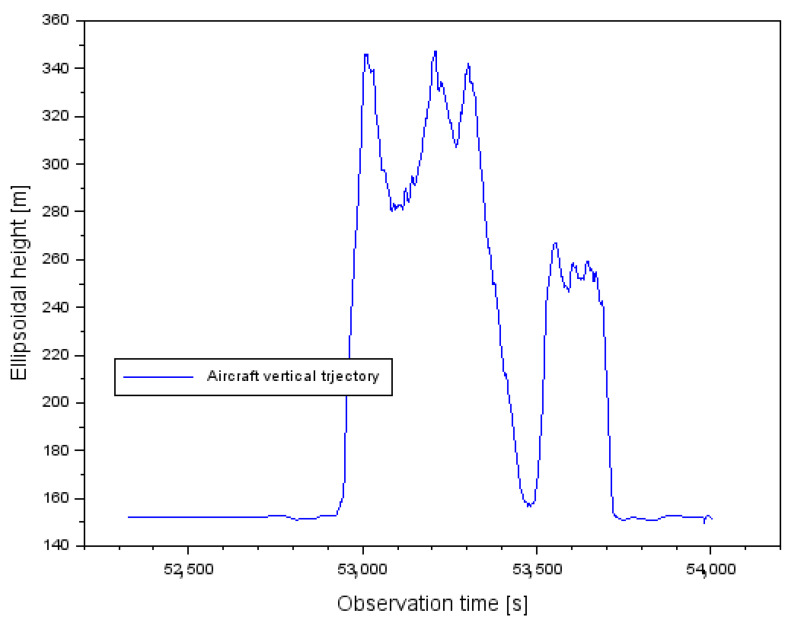
Vertical trajectory of Cessna 172 aircraft.

Figure 6 shows the ellipsoidal altitude values at which the Cessna 172 aircraft was located during the flight. The flight ellipsoidal altitude varied from 149.659 m to 347.374 m. It can be said that the flight ceiling was about 198 m.

During the flight test, the ionosphere state was analyzed using the Global Ionosphere Maps (GIM) model from the Center for Orbit Determination in Europe (CODE) in Switzerland [53]. The resultant value of the VTEC (Vertical TEC) parameter based on the GIM model was 11.8 TECU for the test area. In turn, the RMS (Root Mean Square) accuracy for the VTEC parameter was 0.8 TECU.

The experimental research stage was divided into 2 parts: in the first stage, the position of the Cessna 172 aircraft was determined based on formula (1) for the RTK technique, separately for 3 determinations in the OTF mode. Calculations at this stage were performed in the Trimble Business Center ver.2.70 software [38].

In turn, in the second stage of the research, calculations for the new RTK-OTF positioning strategy were executed in the Scilab v.6.0.0 software [54]. In the source code in the Scilab software, the calculation algorithm for Equations (5)–(17) was implemented. The results of the study are included in Chapter 5 of the paper.

## 5. Results

**Figure 7:**
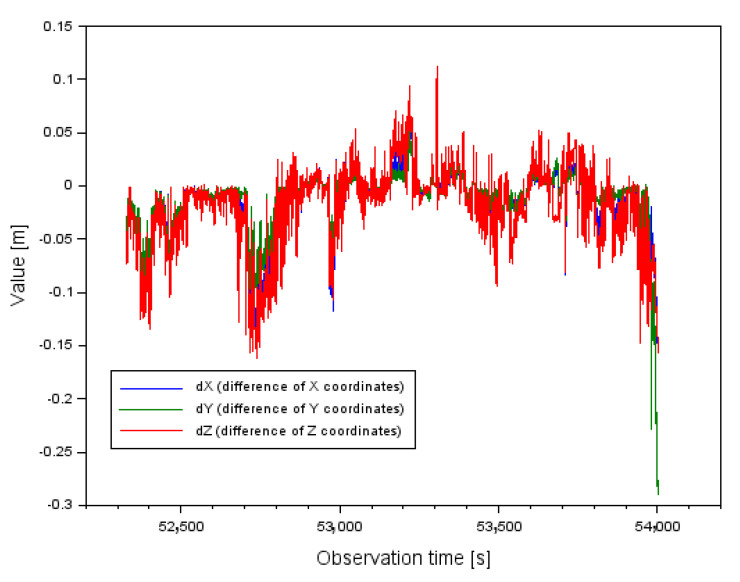
Difference of obtained *XYZ* coordinates of Cessna 172 aircraft.

The presentation of the research results began by presenting the results of the obtained coordinates of the Cessna 172 aircraft for the proposed calculation strategy. Namely, Figure 7 shows the results of the difference of *XYZ* coordinates of the Cessna 172 aircraft from the test method (5–17) for the two measurement weights used. The *XYZ* coordinate difference is respectively:

- from −0.151 m to +0.087 m along the *X* axis,
- from −0.290 m to +0.054 m along the *Y* axis,
- from −0.161 m to +0.112 m along the *Z* axis.

Furthermore, the arithmetic mean for the determined *XYZ* coordinate differences is respectively: −0.016 m along the *X* axis, −0.013 m along the *Y* axis and −0.020 m along the *Z* axis. Therefore, it can be said that the fit of the *XYZ* coordinates from both solutions for the different measurement weights used is relatively high. Looking at the results in Figure 7, one can see an analogous relationship with the parameters shown in Figure 2, Figure 3 and Figure 4. The large jumps in the results of the parameters in Figure 2, Figure 3 and Figure 4 obviously affect the larger difference in *XYZ* coordinates between the solutions for the different measurement weights, which is clearly visible in Figure 7.

**Figure 8:**
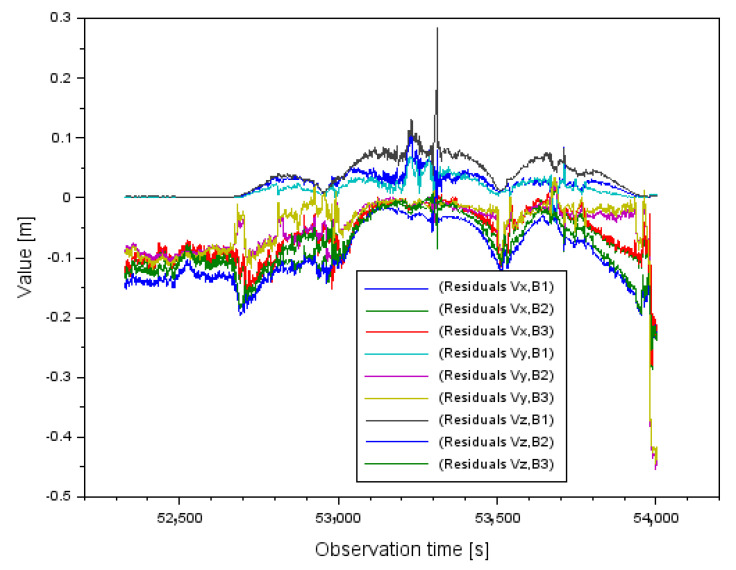
The residuals distribution for case I for measurement weight $P=\frac{1}{d}$.

In the next step, Figure 8 shows the results of the decomposition of the obtained corrections for case I, where the measuring weight $P=\frac{1}{d}$ was used. The correction values are presented separately along the *XYZ* axis. The distribution of the corrections along the *X* axis is from −0.028 m to +0.107 m. In contrast, the distribution of corrections along the *Y* axis ranges from −0.286 m to +0.017 m. The distribution of corrections along the *Z*-axis ranges from −0.281 m to +0.026 m.

**Figure 9:**
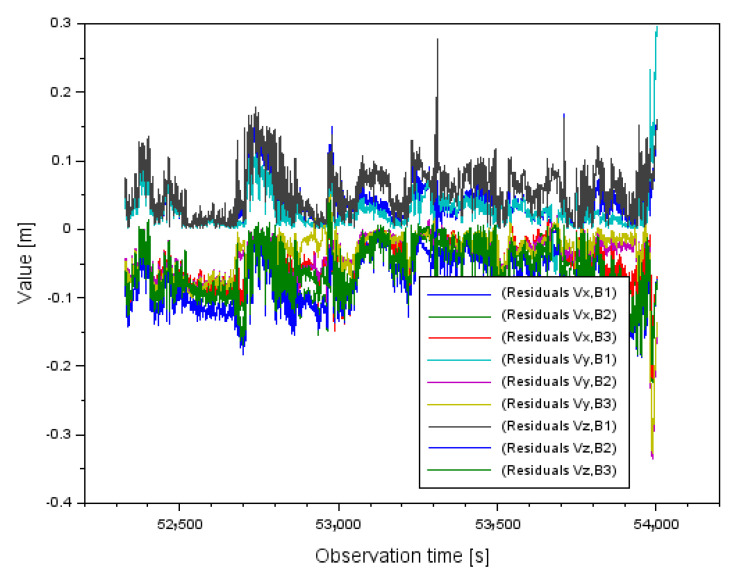
The residuals distribution for case II for measurement weight $P=\frac{1}{md^{2}}$.

In the next step, Figure 9 shows the results of the distribution of the obtained corrections for case II, where the measuring weight $P=\frac{1}{md^{2}}$ was used. The correction values are presented separately along the *XYZ* axis. The distribution of the corrections along the *X* axis ranges from −0.045 m to +0.168 m. In contrast, the distribution of corrections along the *Y*-axis ranges from −0.224 m to +0.011 m and the distribution of corrections along the *Z*-axis ranges from −0.219 m to +0.048 m. In the case of the results in Figure 8 and Figure 9, a relationship can also be observed with respect to the values shown in Figure 2, Figure 3 and Figure 4. Larger values of the corrections occur in measurement epochs where outliers were observed for the mean distance measurement errors or the geometric coefficients of the PDOP.

**Figure 10:**
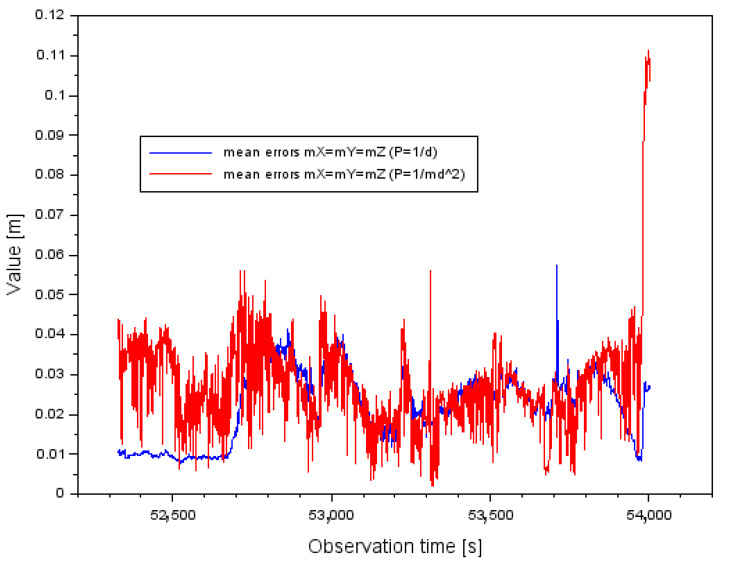
The mean errors for measurement weights $P=\frac{1}{d}$ and $P=\frac{1}{md^{2}}$.

In the presented research method, the measurement weights (see Equations (14) and (15)) are uncorrelated, which has a direct impact on the numerical values of the variance-covariance matrix CQ and the vector of mean errors of the determined coordinates mQ. On this basis, the correlation coefficients defining the relationship between the mean errors in the variance-covariance matrix are 0. Importantly, the results in the analyzed case of mean errors (mX,mY,mZ) will be identical for a given solution with the use of a specific measurement weight. This is also influenced by the construction of the coefficient matrix A, whose numerical values are 0 or 1. Nevertheless, the order of the coefficient matrix A is 3, and the norm of the matrix A is greater than 0. Thus, Figure 10 shows the results of the mean errors (mX,mY,mZ) for a particular solution with different measurement weights. The values of the mean errors (mX,mY,mZ) range from 0.008 m to 0.057 m for a measurement weight of $P=\frac{1}{d}$. On the other hand, the values of the mean errors (mX,mY,mZ) for the measuring weight $P=\frac{1}{md^{2}}$ range from 0.002 m to 0.111 m. It can be said that the scatter of results (mX,mY,mZ) is greater for the measuring weight of $P=\frac{1}{md^{2}}$.

**Figure 11:**
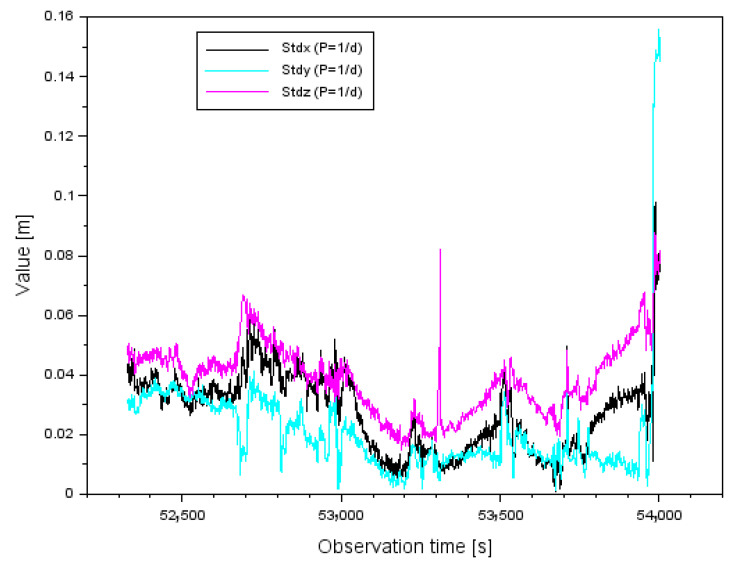
The values of standard deviation for measurement weight $P=\frac{1}{d}$.

The next section of the paper presents the results of the standard deviations (StdX,StdY,StdZ). Figure 11 shows the results of the standard deviations (StdX,StdY,StdZ) for the measuring weight $P=\frac{1}{d}$. The values of the parameter StdX range from 0.001 m to 0.098 m. Furthermore, the arithmetic mean for the parameter StdX is equal to 0.028 m. The values of the parameter StdY range from 0.001 m to 0.156 m. In addition, the arithmetic mean for the parameter StdY is equal to 0.021 m. The values of the parameter StdZ range from 0. 015 m to 0.088 m. In addition, the arithmetic mean for the parameter StdZ is equal to 0.039 m.

**Figure 12:**
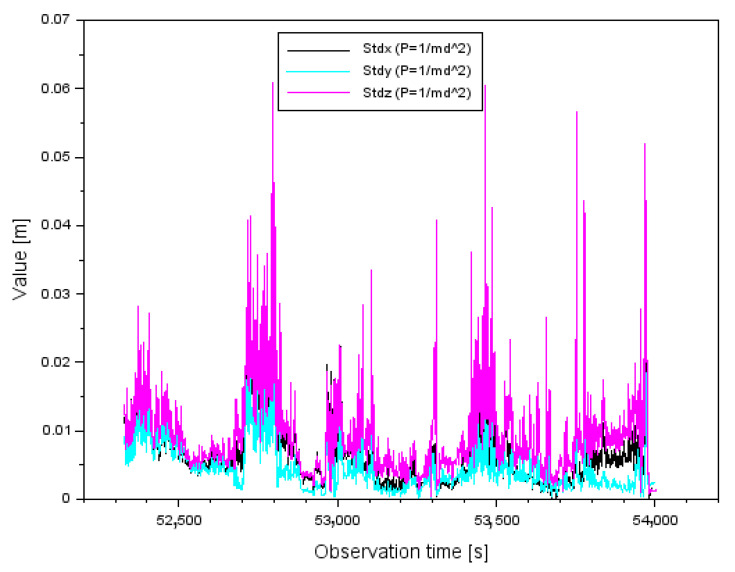
The values of standard deviation for measurement weight $P=\frac{1}{md^{2}}$.

Figure 12 shows the results of the standard deviations (StdX,StdY,StdZ) for the measuring weight $P=\frac{1}{md^{2}}$. The values of the parameter StdX range from 0.001 m to 0.054 m. Furthermore, the arithmetic mean for the parameter StdX is equal to 0.007 m. The values of the parameter StdY range from 0.001 m to 0.036 m. In addition, the arithmetic mean for the parameter StdY is equal to 0.005 m. The values of the parameter StdZ range from 0.001 m to 0.061 m. In addition, the arithmetic mean for the parameter StdZ is equal to 0.009 m.

Comparing the parameter results (StdX,StdY,StdZ) for both measurement weights, it is clear that the standard deviations are smaller for the measurement weight $P=\frac{1}{md^{2}}$. Therefore, the parameter values (StdX,StdY,StdZ) for the measurement weight $P=\frac{1}{md^{2}}$ presented in Figure 12 have been improved by about 75–77% with respect to the results for the measurement weight $P=\frac{1}{d}$ shown in Figure 11. It is worth mentioning that for some single measurement epochs the parameter values (StdX,StdY,StdZ) can be seen as outliers in Figure 11 and Figure 12, which is obviously due to the correction results shown in Figure 8 and Figure 9. The larger the correction value, the standard deviation also increases, which is well shown in Figure 11 and Figure 12.

In the next step of the study, the results of the Chi-square statistical test [55] at the confidence level of 1−α=0.95 and for the f=6 degrees of freedom are shown. Table 2 presents the results of the parameter ∑Pvv and values of the Chi-square $\chi_{f,1-\alpha }^{2}$ test. For the analyzed measurement weights, the values of ∑Pvv are smaller than the $\chi_{f,1-\alpha }^{2}$ values, so the Chi-square statistical test was satisfied for both measurement weights. It can be said that the internal reliability of the computational process has been achieved.

## 6. Discussion

The calculation results show how the proposed research method is effective for the standard solution of the arithmetic mean according to Equation (3). The comparative analysis therefore assesses the accuracy of the determination of the standard deviation parameters (StdX,StdY,StdZ) for the weighted mean model and the arithmetic mean model. In the case of the weighted mean model, the parameters (StdX,StdY,StdZ) were determined according to Equation (16). However, in the arithmetic mean model, the parameters (StdX,StdY,StdZ) were determined as given below:

(18) $$\begin{array}{l} Vx_{B,1}=X_{B,0}-X_{B,1};Vx_{B,2}=X_{B,0}-X_{B,2};Vx_{B,3}=X_{B,0}-X_{B,3}\\ Vy_{B,1}=Y_{B,0}-Y_{B,1};Vy_{B,2}=Y_{B,0}-Y_{B,2};Vy_{B,3}=Y_{B,0}-Y_{B,3}\\ Vz_{B,1}=Z_{B,0}-Z_{B,1};Vz_{B,2}=Z_{B,0}-Z_{B,2};Vz_{B,3}=Z_{B,0}-Z_{B,3}\\ StdX=\sqrt{\frac{\left[Vx_{B,1}\cdot Vx_{B,1}+Vx_{B,2}\cdot Vx_{B,2}+Vx_{B,3}\cdot Vx_{B,3}\right]}{n-1}}\\ StdY=\sqrt{\frac{\left[Vy_{B,1}\cdot Vy_{B,1}+Vy_{B,2}\cdot Vy_{B,2}+Vy_{B,3}\cdot Vy_{B,3}\right]}{n-1}}\\ StdZ=\sqrt{\frac{\left[Vz_{B,1}\cdot Vz_{B,1}+Vz_{B,2}\cdot Vz_{B,2}+Vz_{B,3}\cdot Vz_{B,3}\right]}{n-1}} \end{array}$$

where:

$\left(Vx_{B,1},Vx_{B,2},Vx_{B,3}\right)$—corrections along the *X* axis for the arithmetic mean model,

$\left(Vy_{B,1},Vy_{B,2},Vy_{B,3}\right)$—corrections along the *Y* axis for the arithmetic mean model,

$\left(Vz_{B,1},Vz_{B,2},Vz_{B,3}\right)$—corrections along the *Z* axis for the arithmetic mean model.

**Figure 13:**
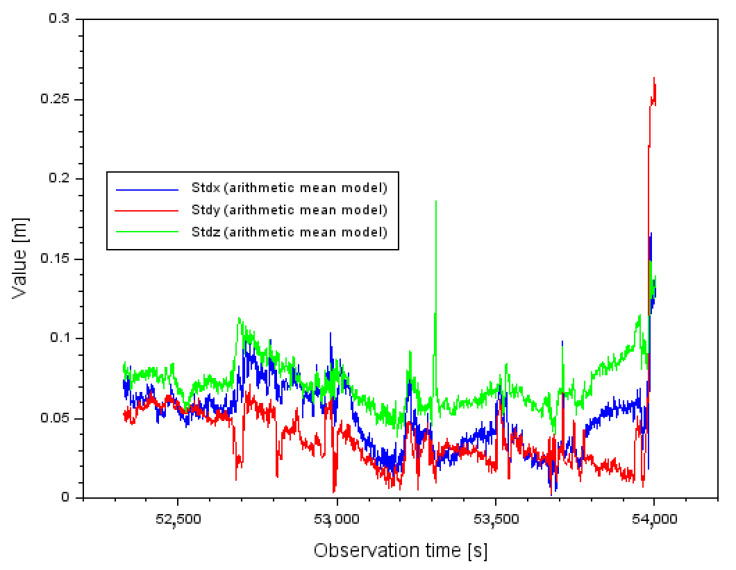
The values of standard deviation based on arithmetic mean model.

Figure 13 shows the parameter results (StdX,StdY,StdZ) obtained from the arithmetic mean model, according to relation (18). The following parameter results were obtained for the arithmetic mean model (StdX,StdY,StdZ):

- The mean value of StdX is equal to 0.051 m and the total spread of results varies between 0.001 m and 0.166 m;
- The mean value of StdY is equal to 0.039 m and the total spread of results ranges from 0.002 m to 0.264 m;
- The mean value of StdZ is equal to 0.072 m and the total spread of results ranges from 0.038 m to 0.186 m.

A comparison was then made between the parameters (StdX,StdY,StdZ) obtained from the arithmetic mean model and the weighted mean model in Table 3. By comparing the standard deviation results (StdX,StdY,StdZ) it can be seen that:

- the parameter values StdX from the weighted mean model $\left(P=\frac{1}{d}\right)$ improved by 45% relative to the results from the arithmetic mean model,
- the parameter values StdY from the weighted mean model $\left(P=\frac{1}{d}\right)$ improved by 46% relative to the results from the arithmetic mean model,
- the parameter values StdZ from the weighted mean model $\left(P=\frac{1}{d}\right)$ improved by 46% relative to the results from the arithmetic mean model,
- the parameter values StdX from the weighted mean model $\left(P=\frac{1}{md^{2}}\right)$ improved by 86% relative to the results from the arithmetic mean model,
- the parameter values StdY from the weighted mean model $\left(P=\frac{1}{md^{2}}\right)$ improved by 87% relative to the results from the arithmetic mean model,
- the parameter values StdZ from the weighted mean model $\left(P=\frac{1}{md^{2}}\right)$ improved by 88% relative to the results from the arithmetic mean model.

Based on the comparative analysis, it can be said that the results of the parameters (StdX,StdY,StdZ) in the weighted mean model have been improved significantly with respect to the solution from the arithmetic mean model.

The numerical analyses carried out in this paper on the application of the RTK-OTF technique in air navigation are very important for several reasons. Firstly, they show which weighting strategy to adopt optimally for the computational process. Secondly, they make it possible to combine individual RTK solutions in OTF mode into a single computational algorithm in a stochastic model. Thirdly, they show the potential user how the weighting model improves the accuracy of the determined coordinates with respect to the weighted mean model. Secondly, they give an answer how to choose parameters for the computational process in the case of the geometry of a three-vector system. The research problem considered in this paper is therefore crucial for the improvement of RTK GPS positioning in air navigation, as highlighted in papers [11,12,13,14,15,16,17,18,19,20,21,22,23,24,25,26,27,28,29,30,31,32,33,34,35,36,37,38,39,40,41,42]. The present work fits into the scope of publications [11,12,13,14,15,16,17,18,19,20,21,22,23,24,25,26,27,28,29,30,31,32,33,34,35,36,37,38,39,40,41,42], where the main objective was to improve RTK GPS positioning in air navigation. As can be seen so far, especially in the scientific studies conducted in Poland [11,29,30,31,32,33,34,35,36,37,38,39,40,41,42], the reference position of the aircraft flight was calculated from the arithmetic mean model of 3 independent GPS RTK solutions in OTF mode. As the results presented in the paper show, the arithmetic mean model can be replaced by an alignment model using measurement weights, which further improves the positioning accuracy. Therefore, the computational algorithm proposed in the paper brings an original solution for determining the reference position of an aircraft flight. Moreover, the applied calculation method can be an interesting alternative to the arithmetic mean model used in works [11,29,30,31,32,33,34,35,36,37,38,39,40,41,42]. It is worth adding that, based on the performed calculations, the proposed calculation algorithm quite significantly reduces the values of the standard deviations of the resultant aircraft position in relation to the results from the arithmetic mean model. This information is crucial from the point of view of the flight reference position determination for the RTK-OTF method in the GPS navigation system.

## 7. Conclusions

The paper presents a new computational strategy for improving the accuracy of aircraft positioning using the RTK-OTF measurement technique in air navigation. Namely, the paper proposes the use of a weighted mean model in a stochastic process to determine the resultant position of an aircraft. The mathematical model of the new solution allows for GPS position alignment on the basis of three independent determinations in the OTF mode. The mathematical model takes into account the measurement weights as a function of the vector length and the mean error of the vector length, respectively. Ultimately, the resultant aircraft coordinates are estimated based on the solution of the least squares method for GPS observations. The proposed research method is a numerical solution using advanced algorithms of the RTK-OTF positioning method.

Real GPS data from the RTK-OTF solution from the flight test performed with the Cessna 172 aircraft for the airport in Dęblin were used in this study. Single RTK solution in OTF mode was calculated in Trimble Business Center 2.70 software, while the final calculations for the resultant aircraft position were performed in Scilab v.6.0.0 software.

The results of the conducted tests show the effectiveness of the proposed solution for determining the accuracy of the RTK-OTF method. Based on the results obtained, the values of standard deviations do not exceed 0.16 m for the measurement weighting as a function of the vector length, and respectively 0.07 m for the measurement weighting as a function of the mean error of the vector length. Weighting the measurements as a function of the mean error of the vector length improves the results of standard deviations by approximately 75–77% compared to the process of weighting as a function of the vector length. Moreover, comparing the obtained results with the classical RTK-OTF solution in the form of the arithmetic mean model, it can be observed that the applied method enables to increase the accuracy of aircraft position determination respectively by 45–46% when using measurement weighting as a function of the vector length, and 86–88% when using measurement weighting as a function of the mean error of the vector length. The obtained results show that the developed method allows to significantly improve the accuracy of the RTK-OTF solution in air navigation. This is extremely important in the context of determining the actual reference position and flight trajectory of an aircraft. In the future, the authors intend to undertake the conducted research with other GNSS navigation systems.

## Figures and Tables

**Table 1:** The summary of research works from scientific knowledge analysis.

Papers from Scientific Knowledge Analysis	Obtained Accuracy	Conclusion
[11,20,22,23,25,29,33,34,36,41,42]	Less than 0.1 m	Concerns mainly the vertical component
[14,15,16,20,22,23,25,28,29,33,34,36,41,42]	Higher than 0.1 m	Concerns mainly the horizontal components or all 3 components (Latitude, Longitude, ellipsoidal height)

**Table 2:** The results of Chi-square test.

Measurement Weight	Values of ∑Pvv	$\mathrm{Statistical}\;\mathrm{Value}\;\mathrm{of}\;\chi_{f,1-\alpha }^{2}$
$P=\frac{1}{d}$	0.001 to 0.077	1.635
$P=\frac{1}{md^{2}}$	0.002 to 1.291	1.635

**Table 3:** The comparison of (StdX,StdY,StdZ) parameters.

Model	Average Value of StdXTerm [m]	Average Value of StdYTerm [m]	Average Value of StdZTerm [m]
Weighted mean model $\left(P=\frac{1}{d}\right)$	0.028	0.021	0.039
Weighted mean model $\left(P=\frac{1}{md^{2}}\right)$	0.007	0.005	0.009
Arithmetic mean model	0.051	0.039	0.072

## Data Availability

The data presented in this study was prepared by Adam Ciećko (University of Warmia and Mazury, Olsztyn).
